# Comparative Analysis of Rumen Microbiota Composition in Dairy Cows with Simple Indigestion and Healthy Cows

**DOI:** 10.3390/microorganisms11112673

**Published:** 2023-10-31

**Authors:** Shuo Wang, Fanlin Kong, Jingjing Liu, Jianmin Xia, Wen Du, Shengli Li, Wei Wang

**Affiliations:** State Key Laboratory of Animal Nutrition, College of Animal Science and Technology, China Agricultural University, Beijing 100193, China; wsss@cau.edu.cn (S.W.); kongfanlin@cau.edu.cn (F.K.); liujing85@cau.edu.cn (J.L.); xjm2020@163.com (J.X.); duwen1022@126.com (W.D.); shenglicau@163.com (S.L.)

**Keywords:** indigestion, rumen, microbiota, health

## Abstract

Simple indigestion in cows leads to substantial economic losses in the dairy industry. Despite ongoing efforts, an effective treatment for this issue remains elusive. Previous studies have emphasized the vital role of rumen microbes in maintaining ruminant health. To deepen our comprehension of the intricate interplay between rumen microbiota and simple indigestion, we undertook a study involving the analysis of rumen fluid from eight cows with simple indigestion and ten healthy cows. Additionally, we collected data pertaining to milk production, rumination behavior, and rumen characteristics. The results showed that cows with simple indigestion displayed significantly lower milk yield, reduced rumination duration, and weakened rumen contraction when contrasted with the healthy cows (*p* < 0.05). However, no significant difference in microbiota α-diversity emerged (*p* > 0.05). Principal coordinate analysis (PCoA) illuminated substantial variations in rumen microbial structure among the two groups (*p* < 0.05). Further analysis spotlighted distinctive bacteria in the rumen of the cows with indigestion, including *Allisonella*, *Synergistes*, *Megasphaera*, *Clostridium_XIVb*, *Campylobacter*, and *Acidaminococcus*. In contrast, *Coraliomargarita*, *Syntrophococcus*, and *Coprococcus* are the dominant bacterial genera in the rumen of healthy dairy cows. Importantly, these key bacterial genera also dominated the overarching microbial interaction network. The observation suggests that changes in the abundance of these dominant bacterial genera potentially underlie the principal etiology of cows with simple indigestion. The present findings can provide insights into simple indigestion prevention and treatment in dairy cows.

## 1. Introduction

Simple indigestion in ruminants, a minor disturbance in GI function, occurs most commonly in cattle and rarely in sheep and goats [1]. It is characterized by complex origins and poor treatment outcomes, resulting in substantial economic losses [2,3,4,5]. Clinically, simple indigestion and subacute ruminal acidosis (SARA) in dairy cows share some similar clinical manifestations, such as reduced appetite, diminished milk production, decreased rumination, and diarrhea [2,6,7]. Consequently, some dairy farmers resort to SARA treatment for rumen indigestion [8]. However, there is a fundamental distinction between simple indigestion and SARA in cows. SARA is defined as a ruminal pH < 5.6 for a duration ranging from 148 to 243 min/d or a ruminal pH < 5.8 for a duration ranging from 284 to 475 min/d, with clinical symptoms often appearing after the stage of pathological low rumen pH [6,7,9]. In contrast, simple indigestion is characterized by rumen motility disorders and microbiota dysregulation, with rumen pH not being its distinctive feature. The primary symptom is weak rumen contractions, and it coincides with the onset of the disease [2,3].

Presently, treatment methods for indigestion remain limited, with the most common approach involving the administration of rumination-promoting drugs through injections and vigorous kneading of the rumen [2]. Rumination-promoting drugs can stimulate parasympathetic nerves, enhancing rumen contractions and gastrointestinal content evacuation. However, this method necessitates precise control of the injection dose and is often associated with side effects [2,10]. Underdosing is ineffective, while overdosing can interfere with rumen movement and overall health. Vigorous kneading of the rumen can also promote rumen contractions and function recovery, but specific kneading duration and intensity remain inconclusive [2]. Additionally, some dairy farmers provide the simple indigestion cows with magnesium hydroxide to stabilize rumen pH [8]. However, the treatment’s efficacy is poor and may lead to systemic alkalization in cows. The reason is that decreased rumen pH is not a clinical feature of cows with simple indigestion. Stephanie et al. discovered that simple-indigestion-afflicted cows exhibit significantly higher rumen pH levels than their healthy counterparts [11]. Therefore, there is an urgent need to develop new methods specifically for treating simple indigestion in dairy cows.

The rumen, an essential digestive organ in ruminant animals, collaborates with other organs such as the intestines and liver to support the animals’ life. In the rumen, numerous microbiota ferment and produce volatile fatty acids, providing energy for ruminants. Hu et al. found that mastitis in dairy cows was caused by ruminal lipopolysaccharide, which enters the bloodstream from the rumen, breaking the blood–milk barrier [12]. Furthermore, Hu et al. also found that a higher abundance of *Stenotrophomonas* in the rumen was closely related to the development of mastitis [12]. Another study revealed that ruminal lipopolysaccharide promotes mastitis by reducing anti-inflammatory enzyme activity in dairy cows [13]. Additionally, Xuan et al. demonstrated that approximately 40% of individual dairy cows’ milk production traits could be explained by the composition and function of rumen microbiota [14]. Moreover, the rumen microbiota was associated with the feed efficiency of dairy cows, with the random forest algorithm effectively predicting feed efficiency based on rumen microbiota, achieving an accuracy of 95.06% [15]. Intriguingly, previous reports suggest that transferring rumen fluid from healthy cows to indigestion-affected cows can effectively alleviate clinical symptoms [16]. Recent studies also reveal that ruminants with SARA can experience swift recovery after being inoculated with rumen fluid from healthy ruminants [17,18,19]. These findings suggest a pivotal role for rumen microbes in determining the rumen health of dairy cows. However, no studies have delved into the comparative rumen microbiota composition between cows with simple indigestion and healthy cows.

Therefore, we hypothesized that there is a notable difference in rumen microbiota composition between healthy and indigestion cows, and that the key microbes may play a pivotal role in the development of indigestion in cows. This study, utilizing the 16S rDNA high-throughput technique, examines the rumen microbiota of eight cows with simple indigestion and ten healthy cows. The aim is to furnish a solid foundation for enhancing the prevention and treatment strategies concerning simple indigestion in cows.

## 2. Materials and Methods

### 2.1. Animal, Management, and Clinical Diagnosis

This study was conducted at a commercial dairy farm in Beijing, China. In the experiment, all dairy cows were housed in free-stall barns, and they could take in the same diet and drink water ad libitum. The diet of the cows was a total mixed ration (TMR, NEL: 7.06 MJ/kg, crude protein: 16.81%, neutral detergent fiber: 29.12%, acid detergent fiber: 19.43%, calcium: 0.86%, phosphorus: 0.33% of DM). TMR was fed three times daily (0700, 1430, and 2200) and was formulated to meet NRC (2001) requirements. In addition, the TMR has a forage-to-concentrate ratio of 6:4, and is free of antibiotics or supplementary additives. Detailed information on the TMR is shown in Table S1. 

This study involved 18 Holstein cows, including 10 healthy cows (healthy group) and 8 cows with simple indigestion (disease group), from one herd. The dairy cows with simple indigestion in a herd (including 182 cows with 81 ± 14 days in milk) was judged by an experienced veterinarian. Initially, dairy cows with recent declines in milk production and rumination duration were screened using dairy cattle management software (Valley Ag Software, Visalia, CA, USA). Rumination duration was generated through the Hr-Tag (a cow collar containing 3-axis accelerometers) (SCR Dairy, Netanya, Israel), and the milk yield of the cows was recorded electronically using BouMatic systems (Boumatic, Madison, WI, USA). The daily recording of rumination duration and milk production for each cow is a routine part of the dairy farm’s production management. Two hours after the morning feeding, the veterinarian observed the cows to assess their mental state and determine if they were showing signs of depression and left undersocket bulging. At the same time, he assessed for clinical symptoms of the rumen through the left flank using the auscultate, palpate, and percussion method. A stethoscope was used to auscultate the clinical symptoms of rumen contractions; palpation was used to check the rumen for firmness; percussion was performed to detect dull resonance in the rumen. Finally, the cows screened as described above underwent rumen pH testing using a portable pH meter (206-pH1, Testo, Shanghai, China), with cows having a pH < 6.0 being excluded, ultimately confirming cases of simple indigestion in the dairy cows.

### 2.2. Rumen Fluid and Data Collection

After identifying the sampled cows, we conducted the sampling on the following day. As described by Han et al. [20], rumen fluid samples from the cows (the total number of samples = 18) were collected in a vacuum syringe via sterilized esophageal tubing before the morning feeding. To ensure the quality of the samples, we initially removed the first 20% of the rumen fluid from the syringe to prevent saliva from interfering with the rumen fluid. Subsequently, a portion of the remaining rumen fluid in the syringe was promptly transferred into cryogenic storage tubes and snap-frozen using liquid nitrogen. Another portion was tested for rumen pH using a portable pH meter (206-pH1, Testo, Germany). Finally, all samples were stored at −80 °C in a refrigerator for subsequent high-throughput sequencing analysis. Two hours after the morning feeding, the veterinarian again auscultated the rumen through the left flank using a stethoscope and recorded the data on contraction strength (weak, medium, strong) and frequency (no./min); the contraction frequency was continuously recorded for 3 min [11]. In addition, we extracted the data on rumination duration and milk production, for the five days prior to sampling the rumen fluid, from the dairy cattle management software for subsequent analysis.

### 2.3. DNA Extraction, PCR Amplification, and 16S rDNA Sequencing

Microbial DNA was extracted from the rumen fluid (18 samples) using PowerSoil DNA Isolation Kit (MoBio Laboratories, Carlsbad, CA, USA) following the manufacturer’s guidelines. The concentration and purity of the extracted DNA were measured with a Nanodrop 2000 spectrophotometer (Thermo Scientific, Waltham, MA, USA). The V3-V4 hypervariable region of the bacterial 16S rDNA gene was amplified with the primers 338F (ACTCCTACGGGAGGCAGCAG) and 806R (GGACTACHVGGGTWTCTAAT). For each sample, a 10-digit barcode sequence was added to the 5′ end of the forward and reverse primers. The PCR was carried out on a Mastercycler Gradient (Eppendorf, Germany) using 25 μL reaction volumes, containing 12.5 μL 2× Taq PCR MasterMix, 3 μL BSA(2 ng/μL), 2 Primer (5 μM), 2 μL template DNA, and 5.5 μL ddH_2_O. Cycling parameters were 95 °C for 5 min, followed by 32 cycles of 95 °C for 45 s, 55 °C for 50 s, and 72 °C for 45 s, with a final extension at 72 °C for 10 min. Three PCR products per sample were pooled to mitigate reaction-level PCR biases. The PCR products were purified using a QIAquick gel extraction kit (QIAGEN, Hilden, Germany), quantified using real-time PCR, and sequenced on an Illumina MiSeq-PE300 platform (Illumina Inc., San Diego, CA, USA), generating 2 × 300 bp paired-end reads.

### 2.4. Sequence Analysis

The obtained paired-end reads in the original DNA fragments were merged using Flash version 1.20 [21], and then each sample was separated according to a unique barcode. After removing barcodes, primers, and splice variants, raw reads were obtained. The raw data were first screened and sequences were removed from consideration if they were shorter than 230 bp, had a low quality score (≤20), contained ambiguous bases, or did not exactly match the primer sequences and barcode tags. Qualified reads were separated using the sample-specific barcode sequences and trimmed with Illumina Analysis Pipeline Version 2.6. Then, the dataset was analyzed using usearch v.8.1. The sequences were clustered into operational taxonomic units (OTUs) at a similarity level of 97% with the uparse method [22] to generate rarefaction curves. The rdp Classifier tool [23] was used to divide representative sequences into different taxonomic groups based on the SILVA ribosomal RNA gene database [24]. 

### 2.5. Statistical Analyses

After the sample number of the lowest sequence was flattened, the MicrobiomeAnalyst platform (https://www.microbiomeanalyst.ca/faces/home.xhtml, accessed on 10 August 2023) was used to analyze the OUT data [25]. The species richness (chao1) and diversity (Shannon) were chosen for alpha-diversity, and the intergroup alpha index variability was demonstrated with the Kruskal–Wallis test. Principal coordinate analysis (PCoA) based on Bray–Curtis dissimilarity matrices was tested using the non-parametric multivariate variance (PERMANOVA) method. The linear discriminant analysis effect size (LEfSe, LDA > 3, *p*-value < 0.05) was used to identify dominant bacteria between the two groups. The heat map clustering plot, stacked bar chart, and network plot were generated using the MicrobiomeAnalyst platform (https://www.microbiomeanalyst.ca/faces/home.xhtml, accessed on 10 August 2023) [25]. The heat map clustering plot was performed based on the genus level; the stacked bar charts were performed based on the phylum and genus level, respectively; the network plot was performed based on the SparCC algorithm (R > 0.6, *p* < 0.05). Furthermore, based on the 16S rRNA data, we predicted ruminal microbiota function of the two groups using the PICRUSt2 software v2.4.1. The results of the predicted ruminal function were analyzed and visualized through the STAMP software v2.1.3 [26]. The PCoA and forest plot were used to show the differences in structure and pathways based on the KEGG level III pathways. Statistical analyses of the duration of rumination and milk yield, as well as rumen characteristic data, were conducted using R software (v 4.0.2). Data were checked for normality using the shapiro.test function of the R software before analysis, and the data fitting non-normality were log transformed. Data on duration of rumination and milk yield were analyzed using the one-way ANOVA method, and the data on the rumen contraction strength were analyzed with the chi-square test. All data are reported as means, and the differences with a *p* < 0.05 were considered statistically significant.

## 3. Results

### 3.1. Apparent Characteristics between Healthy and Simple Indigestion Cows

There are no significant differences in age, parity, and DIM between the two groups of cows. To explore the performance between healthy and simple-indigestion-affected cows, we recorded rumination (min/d) and milk yield (kg/d) for 5 days prior to sampling the rumen fluid. The results show that simple-indigestion-affected cows had lower (*p* < 0.05) rumination duration and milk yield (Table 1). In addition, the experienced veterinarian evaluated the firmness and echo of the rumen as well as rumen contraction strength and frequency. We found that the rumen of the simple-indigestion-affected cows had a firm palpation, and percussion produced dullness (Table 1). At the same time, the healthy cows had higher (*p* < 0.05) rumen contraction strength and frequency than that in the simple-indigestion-affected cows (Table 1). However, there were no significant differences in ruminal pH between the two groups. The results indicated that rumen function is more robust in healthy cows.

### 3.2. Diversity of Ruminal Microbiota 

**Figure 1:**
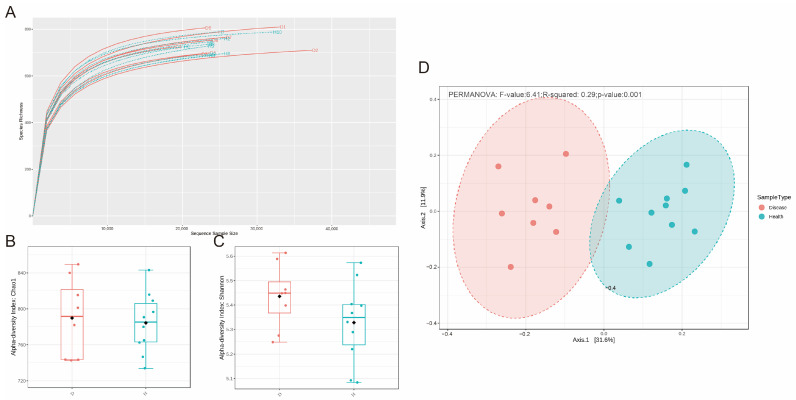
Difference of ruminal microbiota between the healthy and disease groups. (**A**) The rarefaction curve for all samples, (**B**) Chao 1 index of rumen microbiota between the healthy and disease groups, (**C**) Shannon index of rumen microbiota between the healthy and disease groups, (**D**) PCoA plots for the samples based on the Bray–Curtis distance.

A total of 735,290 high-quality 16S rDNA gene sequences were obtained in the rumen samples with an average of 40,849 ± 5413 per sample. The length of the sequences was in the ~360–480 bp range. The sequence coverage was deemed sufficient, as determined by a Good’s coverage greater than 98% for all the samples, and the rarefaction curve gradually became flat with the increase in sequencing depth (Figure 1A). A total of 1264 bacterial OTUs binned at 97% similarity were observed in the dataset.

To furtherly understand the structure of the ruminal microbiota, the Chao1 and Shannon indexes were used to evaluate the richness and diversity based on the OTUs. However, we found that there is no difference in the Chao1 and Shannon indexes between the two groups (*p* > 0.05, Figure 1B,C). The results indicated that cows in the two groups have similar richness and diversity of ruminal microbiota.

Next, we performed principal coordinates analysis (PCoA) based on Bray–Curtis to determine whether the microbial community structure was changed by indigestion. The results showed significant separation of microbiota between the different groups at the OUT level (PERMANOVA, *p* < 0.05, Figure 1D). The results indicated that there is a great difference in the microbial community structure of the rumen between the two groups.

### 3.3. Bacterial Composition of Ruminal Microbiota

**Figure 2:**
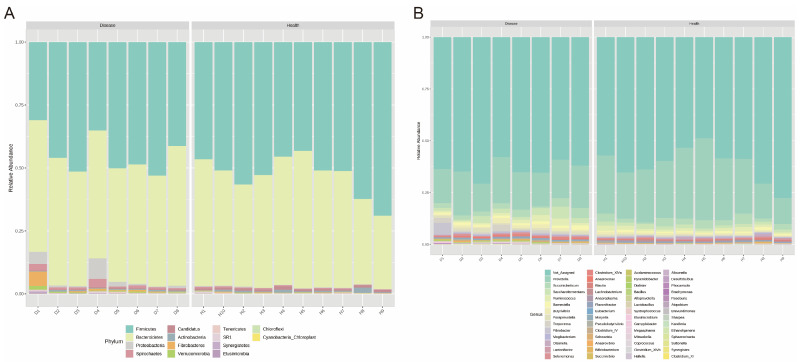
Compositional profiles of rumen microbiota between the healthy and disease groups. (**A**) Phylum level, (**B**) genus level.

Next, we identified the bacterial composition of the samples at the phylum and genus levels. At the phylum level, the relative abundances of rumen bacteria between the two groups were ranked as Firmicutes, Bacteroidetes, Proteobacteria, Spirochaetes, Candidatus, Actinobacteria, Fibrobacteres, Verrucomicrobiota, Tenericutes, SR1, Synergistetes, Elusimicrobiota, and Chloroflexi (Figure 2A); at the genus level, the high relative-abundance genera across the two groups were *Prevotella*, *Succiniclasticum*, *Saccharofermentans*, *Ruminococcus*, *Barnesiella*, *Bamesiella*, *Butyrivibrio*, *Paraprevotella*, *Treponema*, *Fibrobacter*, *Mogibacterium*, *Olsenella*, *Lactonifactor*, *Selenomonas*, *Clostridium_XIVa*, *Anaerovoras,* and so on (Figure 2B). 

**Figure 3:**
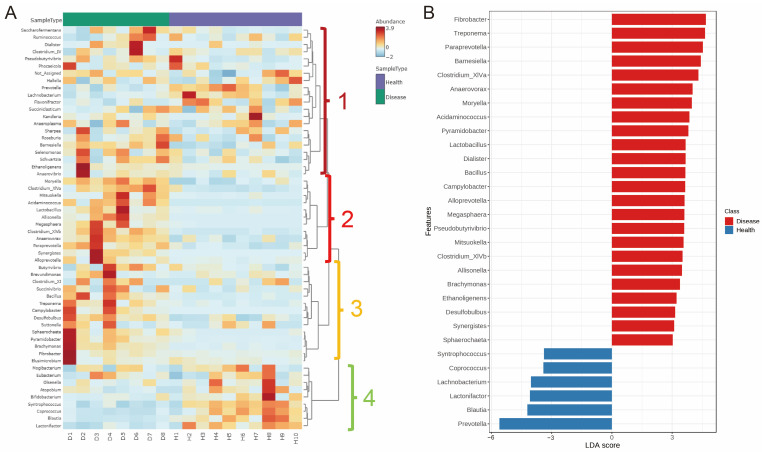
Identification of dominant genera for rumen microbiota between the healthy and disease groups. (**A**) The heat map shows the differences in bacterial genera between the healthy and disease groups based on genus level. Numbers 1, 2, 3, and 4 represent the different clusters, respectively. (**B**) LEfSe analysis of rumen microbiota between the healthy and disease groups based on genus level (LDA > 3, *p* < 0.05).

In addition, we performed a heat map and found four genus clusters. Interestingly, compared with healthy cows, we observed that the indigestion cows had higher genus abundance in clusters 2 and 3 and lower in cluster 4 (Figure 3A). Cluster 2 includes the genera *Mertella*, *Clostridium_XIVa*, *Mitsuokella*, *Acidaminococcus*, *Lactobacillus*, *Allisonella*, *Megasphaera*, *Clostridium_XIVb*, *Anaerovorax*, *Paraprevotella*, *Synergistes*, and *Alloprevotella*; cluster 3 includes *Butyrivibrio*, *Brevundimonas*, *Clostridium_XI*, *Succinivibrio*, *Bacillus*, *Treponema*, *Campylobacter*, *Desulfobulbus*, *Suttonella*, *Sphaerochaeta*, *Pyramidobacter*, *Brachymonas*, *Fibrobacter*, and *Elusimicrobium*; cluster 4 includes *Mogibacterium*, *Eubacterium*, *Olsenella*, *Atopobium*, *Bifidobacterium*, *Syntrophococcus*, *Coprococcus*, *Blautia*, and *Lactonifactor*.

**Figure 4:**
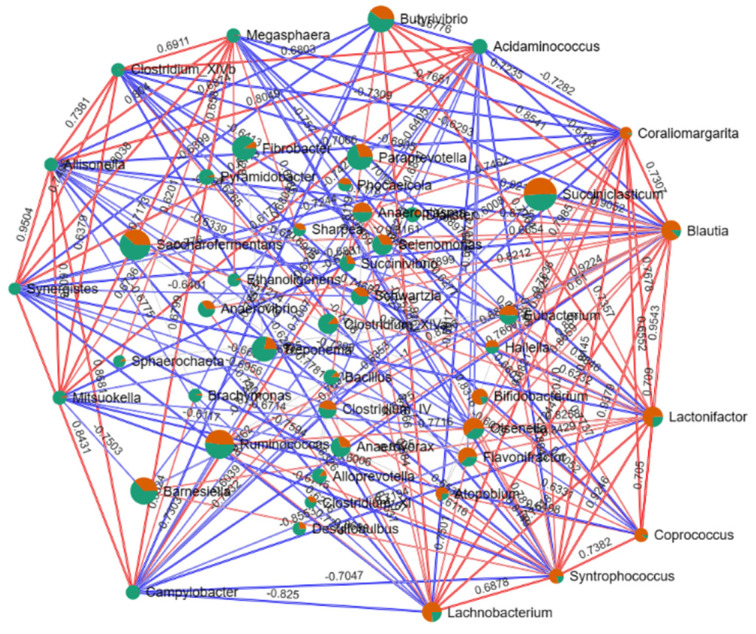
The co-occurring network of genera for the rumen microbiota between the healthy and disease groups. The size of the pie chart represents the relative abundance of genus; the colors in the pie chart represent the groups, with red representing the healthy group and green representing the disease group; the red lines show positive correlations, the blue lines show negative correlations, and the numbers on the lines show the correlation coefficients.

To better understand the dominant genus between the healthy and indigestion cows, we used the LEfSe method. The results showed that the genera *Prevotella*, *Blautia*, *Lactonifactor*, *Lachnobacterium*, *Coprococcus*, and *Syntrophococcus* were dominant in healthy cows; the genera *Fibrobacter*, *Treponema*, *Paraprevotella*, *Barnesiella*, *Clostridium_XIVa*, *Anaerovorax*, *Mortella*, *Acidaminococcus*, *Pyramidobacter*, *Lactobacillus*, *Dialister*, *Bacillus*, *Campylobacter*, *Alloprevotella*, *Megasphaera*, *Pseudobutyrivibrio*, *Mitsuokella*, *Clostridium_XIVb*, *Allisonella*, *Brachymonas*, *Ethanoligenens*, *Desulfobulbus*, *Synergistes*, and *Sphaerochaeta* were dominant in the indigestion cows (Figure 3B). Then, we performed the dominant genus interaction network based on the SparCC method. Shown in Figure 4, the genera *Megasphaera*, *Clostridium*, *Allisonella*, *Synergistes*, *Mitsuokella*, *Campylobacter*, *Lachnobacterium*, *Syntrophococcus*, *Coprococcus*, *Lactonifactor*, *Blautia*, *Coraliomargarita*, *Acidaminococcus*, and *Butyrivibrio* have more complex interactions and are the key bacteria dominating the network. Importantly, these genera are the dominant genus in indigestion and healthy cows, respectively, and the dominant genera in the indigestion cows have a negative relationship with those in the healthy cows.

### 3.4. PICRUSt2 Function Prediction

**Figure 5:**
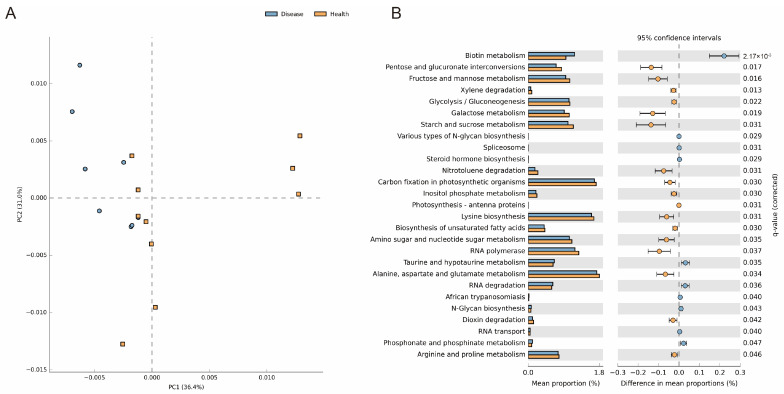
The results of PICRUSt2 function prediction for rumen fluid between the healthy and disease groups. (**A**) PCoA plots for the samples based on the level 3 pathways. (**B**) Functional differences were analyzed using STAMP software based on the level 3 pathways (showing only the significantly different pathways).

Consistent with the distribution of the microbiota, the predicted functions of the rumen microbial communities in the different groups show significant separation in the PCoA plot (Figure 5A). Further, we performed statistical tests at the level 3 pathways (Figure 5B). We found that the pathways including biotin metabolism, various types of N-glycan biosynthesis, spliceosome, steroid hormone biosynthesis, taurine and hypotaurine metabolism, RNA degradation, African trypanosomiasis, N-Glycan biosynthesis, RNA transport, and phosphonate and phosphinate metabolism significantly up-regulate in indigestion cows, while the pathways including pentose and glucuronate interconversions, fructose and mannose metabolism, glycolysis/gluconeogenesis, starch and sucrose metabolism, carbon fixation in photosynthetic organisms, lysine biosynthesis, alanine, aspartate and glutamate metabolism, arginine and proline metabolism, and so on, significantly up-regulate in healthy cows. The results indicate that there is a severe reduction in the metabolic capacity of the indigestion cows. 

## 4. Discussion

Simple indigestion in cows poses a significant challenge to the dairy industry, leading to substantial economic losses. Currently, the primary clinical approach involves administering agents to enhance rumen motility. However, concerns persist regarding its effectiveness and potential side effects. To address these issues, our study compares the ruminal composition between healthy and simple-indigestion-affected cows, pinpointing the dominant bacteria that could provide a foundation for developing clinical treatments for cows with simple indigestion.

Accurate clinical judgment of cow diseases is crucial, as it directly impacts the relevance of experimental results. Given that many cow diseases share similar clinical presentations, multiple criteria are needed for combined validation to determine the specific disease. Some studies have confirmed that the average daily rumination duration for healthy cows fed a TMR diet exceeds 450 min/d, and their ruminal pH remains between 6 and 6.5 [27,28,29,30]. In this study, the average daily rumination time for healthy cows (561 ± 62) min/d and rumen pH (6.22 ± 0.3) were both within the normal range, indicating that the healthy cow samples in this study are highly representative. Similarly, previous research related that cows with simple indigestion exhibited lower rumination time and yield, as well as weaker rumen contractions [11]. The clinical symptoms of those cows are consistent with the indigestion cows in our study. Notably, the ruminal pH of the diseased cows was >5.6 and showed no significant difference from that of the healthy cows. These results affirm that the cows with indigestion in our study were not affected by SARA. Subsequently, we examined the rumen microbiota of both healthy and simple-indigestion-affected cows. While the diversity did not show significant differences, marked variations in richness and structure were observed between the two groups. The results align with our hypothesis, indicating that the simple-indigestion-affected cows possess a distinct rumen microbiota. Compared with healthy cows, the abundance of Firmicutes and Candidatus exhibited a decrease, while the abundance of Proteobacteria, Spirochaetes, Verrucomicrobia, Fibrobacteres, and Synergistetes showed an increase in the simple-indigestion-affected cows. Firmicutes are known to break down and ferment fibers in the rumen, producing acetic acid and butyric acid for ruminant use [20,31]. Proteobacteria can promote nitrogen circulation in the rumen [32,33], and Fibrobacteres can produce cellulase, aiding in cellulose breakdown [34]. These results suggest that the rumen environment of the diseased cows may have become imbalanced, resulting in nutrient digestion problems. Moreover, previous research has shown that high-concentrate diets reduce Firmicutes and Verrucomicrobia while increasing Bacteroides in lamb rumens [35], which showed a difference from the results of this study. This discrepancy further emphasizes the essential difference between cows with simple indigestion and SARA from a rumen microorganism perspective.

Recent studies have revealed a complex network of relationships among microbes that govern microbiome functions through intricate quorum sensing mechanisms [36]. Importantly, within these complex network relationships, one or several key microbes dominate the construction and stability of the entire network. Interestingly, our study results also identify certain key genera that dominate entire microbial interaction networks. In cows with simple indigestion, the genera *Allisonella*, *Synergistes*, *Megasphaera*, *Clostridium_XIVb*, *Campylobacter*, and *Acidaminococcus* stand out, while the genera *Coraliomargarita*, *Syntrophococcus*, and *Coprococcus* dominate in healthy cows. Importantly, positive correlations exist within each group’s key bacteria, while negative correlations occur between the indigestion and healthy cows. These findings imply that these bacteria play a vital role in the development of indigestion in cows. *Allisonella* was found in cattle and horse laminitis, and it can produce histamine [37]. Histamine has a strong vasodilatory effect, increasing the permeability of the capillary and venule walls and leading to local tissue edema [38,39]. *Synergistes* is often linked to intestinal and soft tissue infections [40,41]. In addition, recent studies have found that *Synergistes* appears to be more prevalent in people with tooth and gum disease than in healthy people [42,43]. Both *Clostridium* and *Campylobacter* are associated with digestive tract disorders, with the former releasing an endotoxin causing mucosal necrosis [44,45] and the latter causing enteritis [46]. The increased abundance of these genera also suggests that inflammation may be present in the rumens of cows with indigestion. Interestingly, the most important function of *Synergistes* is the degradation of amino acids [41]. *Acidaminococcus* thrives on amino acids, especially glutamic acid [47]. These results imply that indigestion cows may be deficient in amino acids. *Syntrophococcus* is isolated from the rumens of hay-fed cattle, and acetic acid is its only product [48]. Additionally, Hao et al. found that compared with the pre-weaning period, *Syntrophococcus* is a key genus in post-weaning calves [49]. After weaning, the nutrient source of the calves was changed from milk or milk replacer to solid feed, and the main digestive organ was changed from the intestine to the rumen. Therefore, we believe that *Syntrophococcus* plays an important role in the establishment and improvement of rumen function. *Coprococcus*, generally beneficial for animals, enhances hosts’ microbial balance, boosts disease resistance, and produces antimicrobial compounds [50]. We believe that these dominant bacteria genera are the key to maintaining rumen homeostasis and are worthy of further study. 

Furthermore, changes in rumen microbial abundance can lead to variations in rumen microbiota function. The function of the microbiome is critical, as it directly affects the host’s metabolism and health [51,52]. In this study, we observed up-regulated pathways related to lysine synthesis, carbohydrate metabolism, and gluconeogenesis, along with downregulated pathways related to biotin and hormone metabolism in the rumens of healthy dairy cows. Lysine, being the first limiting amino acid in dairy cows, can significantly improve milk production performance and enhance immune function when supplemented [53,54,55]. Arginine and glutamate, as functional amino acids, have been shown to relieve dairy stress by regulating immunity and inflammation [56]. Similarly, the down-regulation of carbohydrate metabolism pathways in the rumen of simple-indigestion-affected cows further suggests the feasibility of regulating rumen microbiota to alleviate indigestion symptoms in dairy cows. The up-regulation of biotin and hormone metabolic pathways in indigestion-affected cows may indicate a self-protective regulation mechanism between dairy cows and rumen microorganisms. Biotin can stimulate rumen contractions and expedite chyme excretion. In summary, the PICRUSt2 results corroborate our previous speculations. These findings further suggest that cows with indigestion might experience nutritional deficiencies due to their unbalanced rumen homeostasis.

In summary, we propose that simple-indigestion-affected cows undergo unbalanced rumen homeostasis resulting in nutrient deficiency and inflammation. This suggests that the treatment of cows with simple indigestion should involve not only antibiotics but also supplemental nutrients such as amino acids. However, our study provides only preliminary insights into rumen microbial differences and key microbes in varying cow states. Further exploration using multi-omics approaches in conjunction with data such as feed intake is essential to unraveling related metabolic mechanisms, ultimately contributing to the development of effective prevention and treatment strategies for cows with simple indigestion.

## 5. Conclusions

This study conducted a comprehensive comparison of the bacterial composition in the rumens of cows with simple indigestion and healthy cows, revealing significant differences in the microbial composition. Notably, rumen *Allisonella*, *Synergistes*, *Megasphaera*, *Campylobacter*, *Acidaminococcus*, *Syntrophococcus*, and *Coprococcus* are key bacteria genera that may cause simple indigestion in dairy cows. These findings offer valuable insights for the prevention and treatment of rumen indigestion in dairy cows. However, further research is essential to validate the roles of these identified bacteria and understand their interactions with their host organisms.

## Figures and Tables

**Table 1:** Physical examination variables for 8 cows with simple indigestion and 10 healthy cows.

Items	Disease	Health	*p*-Value
Age (years)	5.2 ± 0.58	4.9 ± 0.81	0.390
DIM ^1^ (days)	81 ± 2.12	80 ± 2.36	0.366
Parity	3.25 ± 0.89	2.90 ± 0.57	0.324
Ruminal pH	6.13 ± 0.4	6.22 ± 0.3	0.592
Duration of rumination (min/d)	324 ± 95	561 ± 62	<0.05
Milk yield (Kg/d)	32.7 ± 6.2	47.1 ± 7.7	<0.05
Firmness ^2^ (Left rumen)	Yes	No	
Dullness ^3^ (Rumen)	Yes	No	
Rumen contraction strength ^4^	1	2.5	<0.05
Rumen contraction frequency (No./min)	1.21 ± 0.7	2.34 ± 0.3	<0.05

Values represent the mean ± SD (standard deviation). ^1^ DIM = days in milk; ^2^ results of palpation of the left rumen; ^3^ percussion results of the left rumen; ^4^ rumen contraction strength as assessed on a 3-point scale (1 = weak, 2 = moderate, and 3 = strong) [11].

## Data Availability

The 16S rDNA gene sequencing reads were deposited in the Genome Sequence Archive1 in the BIG Data Center under the accession number: PRJCA000874.

## Supplementary Materials

The following supporting information can be downloaded at: https://www.mdpi.com/article/10.3390/microorganisms11112673/s1, Table S1: The composition and nutritional value of the TMR diet.
