# Supplementary material for: Comparative Analysis of Rumen Microbiota Composition in Dairy Cows with Simple Indigestion and Healthy Cows
Source: Microorganisms. 2023 Oct 31;11(11):2673. doi: 10.3390/microorganisms11112673 (PMC10672840; doi:10.3390/microorganisms11112673)
Supplement: Supplementary file 1 [file microorganisms-11-02673-s001.zip › microorganisms-2589652-supplementary.pdf]

Table S1. Ingredients and chemical composition of the total mixed ration

Item	Content
Ingredients	(% of feeding basis)
Corn silage	45.26
Alfalfa	13.76
Corn	1.4
Soybean meal	8.36
Extrusion Soybean	1.5
Flaked corn	14.64
Soybean hull	8.25
Cotton seed	2.8
Premix[1)]	4.03
Total	100
Nutrient levels[2)]	(% dry matter)
NEL/(MJ/kg)	7.06
CP	16.81
NDF	29.12
ADF	19.43
Ca	0.86
P	0.33

1) One kilogram of complete diet (on dry matter basis) contained the following minerals and vitamin premix: Mn, 4,800 mg; Fe, 4,800 mg; Zn, 12,850 mg; Cu, 3,250 mg; I, 140 mg; Se, 150 mg; Co, 110 mg; Vitamin A, 1,000,000 IU; Vitamin D_3, 280,000 IU; Vitamin E, 10,000 IU; niacin, 1,000 mg.

2) NEL values were calculated using the net energy of lactation values of feedstuffs from NRC (2001); others were measured by laboratory analysis of the total mixed ration.
